# The interaction between HIV testing social norms and self-efficacy on HIV testing among Chinese men who have sex with men: results from an online cross-sectional study

**DOI:** 10.1186/s12879-018-3454-5

**Published:** 2018-10-30

**Authors:** Peizhen Zhao, Li Liu, Ye Zhang, Huanhuan Cheng, Bolin Cao, Chuncheng Liu, Cheng Wang, Bin Yang, Chongyi Wei, Joseph D. Tucker, Weiming Tang

**Affiliations:** 1 Dermatology Hospital, Southern Medical University, Guangzhou, China; 2 Nanjing Municipal Center for Disease Control and Prevention, Jiangsu, China; 3 The Third Affiliated Hospital, Sun Yat-Sen University, Guangzhou, China; 4 SESH study group of University of North Carolina at Chapel Hill, Guangzhou, China; 5 University of North Carolina at Chapel Hill Project-China, Guangzhou, 510095 China; 6 Social and Behavioral Health Sciences, School of Public Health, Rutgers University, Piscataway, NJ USA; 7 School of Medicine of University of North Carolina at Chapel Hill, Chapel Hill, USA

**Keywords:** Men who have sex with men (MSM), Social norm, Self-efficacy, HIV testing

## Abstract

**Background:**

Increasing human immunodeficiency virus (HIV) testing is critical for HIV control. This study aimed to evaluate the interaction between social norms and self-efficacy on HIV testing among Chinese men who have sex with men (MSM).

**Methods:**

We conducted an online survey in eight Chinese cities in Shandong and Guangdong Provinces in July 2016. We included participants who were born as a male, at least 16 years old, currently living in one of the designated cities, and had ever engaged in anal sex with a man. We collected information regarding socio-demographics, high-risk behaviors, and history of HIV and other STI testing. We coded sensitivity to social norms using six items asking participants about their perceived social norm regarding HIV testing. We coded HIV testing self-efficacy using a separate six-item scale. We interpreted higher mean scores as higher sensitivity to social norms and higher self-efficacy, respectively. We conducted logistic regressions to evaluate the interaction between self-efficacy and social norms on HIV testing.

**Results:**

A total of 2105 men completed the survey. The mean age of the participants was 25.97 ± 6.42 years. Over four-fifths (85.9%) of participants were unmarried, 22.7% were students, and 64.6% at least had a college degree. 62.5 and 32.6% of participants ever and tested HIV in the last three months, respectively.

With respect to uptake of HIV testing in the last three months, the adjusted odds ratio was 1.01(95% CI: 0.96–1.06) for higher sensitivity to social norms and 1.09 (95% CI: 1.05–1.14) for higher self-efficacy, with an interaction effect of 1.02 (95% CI: 1.01–1.03), respectively. With respect to uptake of lifetime HIV testing, the adjusted odds ratio was 1.03(95% CI: 0.99–1.07) for higher sensitivity to social norms and 1.15 (95% CI: 1.11–1.19) for higher self-efficacy, with an interaction effect of 1.02 (95% CI: 1.01–1.04), respectively.

**Conclusions:**

Our survey demonstrated that there is a significant association between the uptake of HIV testing with sensitivity to the social norm, higher self-efficacy, as well as the interaction between them. Tailored studies for improving HIV testing among MSM in China can combine these two interventions together.

**Electronic supplementary material:**

The online version of this article (10.1186/s12879-018-3454-5) contains supplementary material, which is available to authorized users.

## Introduction

Men who have sex with men have become a key population for HIV infection [1]. Increasing the uptake of HIV testing among MSM is a crucial component of the HIV treatment cascade and the control of the global HIV epidemic [2].

To scale up HIV testing services, the Chinese government has established voluntary counseling and testing (VCT) clinics that offer free HIV testing services and provider-initiated testing and counseling (PITC) [3], but only 60% of Chinese men who have sex with men (MSM) have ever tested for HIV [4]. Among Chinese MSM, potential reasons for low uptake of HIV testing include stigma against HIV, limited access to HIV testing service, and poor sexual orientation disclosure [3, 5, 6]. Identifying potential strategies to reduce these barriers and further improving HIV testing uptake among Chinese MSM is essential.

Studies in China and other countries indicated that HIV testing social norm and self-efficacy are two important psychosocial factors can facilitate HIV testing among MSM [7–9]. Self-efficacy refers to the belief in one’s own ability to complete tasks and reach goals [8]. According to the social cognitive theory, self-efficacy plays an important role in the adoption, initiation, and maintenance of health behaviors [8]. HIV testing self-efficacy refers to people’s level of confidence to have HIV testing [10]. Social norms are social attitudes of approval or disapproval that specify what ought and ought not to be done and are significant in the context of health [11]. HIV testing social norms refer to people’s social attitudes about HIV testing [11]. Improving HIV testing self-efficacy and perceived positive social norms of HIV testing are two intervention strategies for improving uptake of HIV testing among MSM [8, 9, 12].

Even HIV testing social norm and self-efficacy has a positive impact on promoting HIV testing, whether these two can interact with each other and further strengthen the intervention effect is still not clear while knowing this is critical for designing tailored interventions for MSM. Therefore, the aim of this study was to examine whether there exists an interaction effect between perceived HIV testing-related social norm and self-efficacy on HIV testing among Chinese MSM. The hypothesis of this study is that the perceived HIV testing-related social norm and self-efficacy can interact with each other and further improve HIV testing uptake.

## Methods

### Study population

A nationwide cross-sectional online survey among MSM was conducted in eight Chinese cities: Guangzhou, Shenzhen, Zhuhai, Jiangmen (Guangdong Province, Southern China), Jinan, Qingdao, Jining, and Yantai (Shandong Province, Northern China) in July 2016. These eight cities of Guangdong and Shandong Province were chosen because they were urban cities with the relatively high prevalence of HIV.

We recruited participants online by collaborating with a gay dating application. Banner advertisements with links to the online survey were sent to registered users of the application. Viewers who clicked the survey link were directed to the web survey.

We only included participants who were born biologically as a male, at least 16 years old, and currently living in one of the designated cities who had had anal sex with a man at least once during their lifetime. We also required participants to provide their cell phone numbers for other follow-up purposes and agree to informed consent. All eligible participants received a small phone card reimbursement or WeChat incentive transfer (equivalent to roughly 7.5 USD).

### Measures

After screening for eligibility and signing the informed consent, we first collected information on socio-demographic information, including age (which we further categorized into three groups: less than 20, 20–29, or 30 and above), marital status (never married or ever married, including widowed or divorced), occupation (student or not), education (senior high school or below, college/bachelors, or masters or PhD), and monthly income (less than 250 USD, 250–500 USD, 501–800 USD, 801–1250 USD or above 1250 USD). We also asked participants whether they had ever been tested for HIV (including HIV self-testing and facility-based testing) in their lifetime, whether ever self-tested, and whether testing for HIV in the last 3 months. HIV testing in lifetime was measured by asking participants whether they performed any HIV testing in their lifetime, including testing in general hospitals, clinics, the point of care sites or HIV self-testing.The HIV self-testing was defined as people tested HIV in private and interpreted the results on their own [13]. HIV testing in the past 3 months was measured as whether the participants performed any HIV testing in the last 3 months.

### HIV testing self-efficacy

HIV testing self-efficacy was measured using six questions that elicited participants’ attitudes toward and confidence in receiving HIV testing [14, 15]. The items are shown in Table 2. All items were scored using a 4-point Likert-type response set, with answers ranging from strongly agree (4) to strongly disagree (1). For each participant, a mean score was calculated with 4 as the highest possible score of 4 and 1 as the possible lowest score. Higher mean scores indicated higher HIV testing self-efficacy. The scale assessing respondents’ overall levels of HIV testing self-efficacy was found to be reliable (Cronbach’s *α* = 0 .792).

### HIV testing social norm

Social norm was measured by six items asking participants about their awareness of social norms about HIV testing [11, 16] (see Table 2). All items were scored using a 4-point Likert-type response set, with answers ranging from strongly agree (4) to strongly disagree (1). For each participant, a mean score was calculated with 4 as the highest possible score of 4 and 1 as the possible lowest score. Higher mean scores indicated higher sensitivity to HIV testing social norms. The Cronbach’s *α* of HIV testing social norm was 0.695.

### Statistical analysis

We used descriptive analysis to describe socio-demographics, high-risk behaviors, and self-efficacy among the participants who had been tested for HIV in the past three months and those who had not. We also analyzed the linear assumption through semiparametric regression map (Additional file 1). Spearmen correlation tests were used to identify any association between social norm and self-efficacy. Univariate and multivariable logistic regressions were used to evaluate the association between HIV testing and self-efficacy and HIV testing and sensitivity to social norms. We used a logistic regression multiplication model to analyze the association of the interaction between HIV testing social norm and HIV testing self-efficacy. We used multivariable logistic regression to analyze the influencing factors of HIV testing. Age, education, marital status, and income were a covariate in the model. These variables were chosen based on our prior knowledge, and a directed acyclic graph (DAG) [17] was drawn to assist in this analysis. Throughout all the analyses, results are reported as statistically significant whenever *P* < 0.05. All data were analyzed using SAS 9.4 (SAS Int. Cary, NC, USA).

## Results

### Socio-demographic characteristics and behaviors

Two thousand one hundred five participants finished the online survey. The mean age of participants was 26.0 ± 6.4 years. 54.4% of the participants were ≤ 25 years old, 14.1% were married, 22.7% were students, 64.6% had a college or above degree, and 71.6% had an annual income less than $9700 USD (60,000 RMB). Also, 72.4% of the participants self-identified as gay, and 23.6% self-identified as bisexual (Table 1).

**Table 1:** Sociodemographic characteristics of MSM participants (*n* = 2105)

Characteristics	Frequency (*N* = 2105)	Percentage (%)
Age^a^		
≤ 25	1146	54.44
26–35	789	37.48
36–45	149	7.08
≥ 46	21	1.00
Student		
Yes	477	22.66
No	1628	77.34
Marital status		
Not married	1809	85.94
Engaged or Married	187	8.88
Separated or Divorced	106	5.04
Widowed	3	0.14
Education level		
High school or below	746	35.44
College diploma	583	27.70
Undergraduate	697	33.11
Postgraduate (Master/PhD)	79	3.75
Individual monthly income		
<1500 RMB	391	18.57
1500–3000 RMB	425	20.19
3001–5000 RMB	690	32.78
5001–8000 RMB	384	18.24
>8000 RMB	215	10.21
Gender identity		
Male	1999	94.96
Female	33	1.57
Transgender	34	1.62
Unsure/Other	39	1.85
Sexual orientation		
Homosexual	1524	72.40
Bisexual	496	23.56
Heterosexual	11	0.52
Unsure/Other	74	3.52
HIV testing in lifetime		
Yes	1315	62.5
No	790	37.5
HIV Testing in the past 3 months		
Yes	687	32.6
No	1418	67.4
HIV self-testing in lifetime		
Yes	685	32.5
No	1420	67.5

^a^Age: mean = 25.97, SD = ±6.42

### HIV testing

Among 2105 participating MSM, 1315 (62.5%) reported being tested for HIV at least once in their lifetime. However, only 687(32.6%) participants had been tested for HIV within the past 3 months. Moreover, 685(32.5%) participants reported having self-tested for HIV at least once in their lifetime.

### Social norm and self-efficacy

In this study, the median(interquartile range, IQR) score of HIV testing social norms was 17.0 (15.0–18.0). While around 75% of participants endorsed all the statements of HIV testing social norms, between 17.2 and 52.2% of participants did not endorse each individual item. For HIV testing self-efficacy, the median (IQR) score was 19.0 (17.0–21.0). A majority of participants endorsed these statements, while a small proportion (2.8–26.8%) did not endorse individual items. (Table 2).

**Table 2:** Distribution of responses for items related to social norms and self-efficacy

	Strongly agree	Agree	Disagree	Strongly disagree
Social Norm				
Most gay men want to get tested but are afraid to get tested.	513(24.37)	977(46.41)	517(24.56)	98(4.66)
Most gay men who get tested do not want others to find out they were tested.	746(35.44)	969(46.03)	341(16.20)	49(2.33)
Most gay men want to get tested for HIV.	762(36.20)	981(46.60)	345(16.39)	17(0.81)
Most gay men who want to get tested will tell their partners they want to get tested	490(23.28)	995(47.27)	575(27.32)	45(2.14)
Most gay men have been tested for HIV	323(15.34)	684(32.49)	968(45.99)	130(6.18)
Most gay men get tested for HIV only if they are sick or feel uncomfortable	393(18.67)	968(45.99)	634(30.12)	110(5.23)
Self-Efficacy				
You would feel comfortable discussing HIV testing with a potential partner	508(24.13)	1033(49.07)	495(23.52)	69(3.28)
You feel confident that you could refuse to have sex with a partner who did not want to undergo HIV testing	686(32.59)	860(40.86)	447(21.24)	112(5.32)
You feel confident that you could persuade your partner to undergo HIV testing	717(34.06)	1152(54.73)	224(10.64)	12(0.57)
You can get tested for HIV if you wish	1004(47.70)	1043(49.55)	52(2.47)	6(0.29)
You would get tested for HIV even if you are afraid to know the results	925(43.94)	1074(51.02)	91(4.32)	15(0.71)
You have confidence that you will undergo HIV testing regularly	541(25.70)	1028(48.84)	501(23.80)	35(1.66)

### The association between HIV testing with sensitivity to social norms and self-efficacy

Univariate analysis indicated that MSM with higher sensitivity to social norms had a higher proportion of HIV testing in the past 3 months, though the result was not significant (Crude odds ratio (cOR, main effect) = 1.02 [0.96–1.06]). Univariate analysis also indicated that MSM with higher self-efficacy had more HIV testing in the past 3 months, with a cOR (main effect) of 1.09 [1.05–1.13]. We also detected an association of the interaction between sensitivity to HIV testing social norms and self-efficacy with HIV testing in the last 3 months (cOR = 1.02 [1.01,1.04], see Table 3).

**Table 3:** The association of HIV testing with Social Norms, Self-Efficacy, and their interaction

	Crude Model				Adjusted Model*			
	Coefficient	*SE*	*OR* (95% CI)	*P*-value	Coefficient	*SE*	*OR* (95% CI)	*P*-value
HIV Testing in the past 3 months								
Intercept	−0.16	0.42	–	–	0.82	0.51	–	–
Social norm	0.01	0.02	1.02(0.96,1.06)	0.55	0.01	0.02	1.01(0.96,1.06)	0.75
Intercept	−1.58	0.40	–	–	−0.71	0.48	–	–
Self-efficacy	0.09	0.02	1.09(1.05,1.13)	**< 0.001** ^**#**^	0.09	0.02	1.09(1.05,1.14)	**< 0.001** ^**#**^
Intercept	5.10	2.88	–	–	5.47	2.92	–	–
Social norm	−0.40	0.17	0.67(0.48,0.93)	**0.018** ^**#**^	−0.38	0.17	0.69(0.49,0.96)	**0.029** ^**#**^
Self-efficacy	−0.23	0.14	0.79(0.60,1.05)	0.11	−0.20	0.15	0.82(0.62,1.09)	0.17
Social norm*Self-efficacy	0.02	0.01	1.02(1.01,1.04)	**0.023** ^**#**^	0.02	0.01	1.02(1.01,1.03)	**0.042** ^**#**^
HIV testing in lifetime								
Intercept	0.24	0.35	–	–	−2.20	0.43	–	–
Social norm	0.02	0.02	1.02(0.98,1.06)	0.42	0.03	0.02	1.03(0.99,1.07)	0.20
Intercept	−2.07	0.31	–	–	−4.42	0.41	–	–
Self-efficacy	0.14	0.02	1.15(1,11,1.89)	**< 0.001** ^**#**^	0.14	0.02	1.15(1.11,1.19)	**< 0.001** ^**#**^
Intercept					−12.14	2.55	–	–
Social norm	0.39	0.14	1.41(1.10,1.80)	**0.007** ^**#**^	0.44	0.15	1.48(1.14,1.92)	**0.003** ^**#**^
Self-efficacy	0.54	0.12	1.64(1.32,2.04)	**< 0.001** ^**#**^	0.58	0.13	1.70(1.36,2.13)	**< 0.001** ^**#**^
Social norm*Self-efficacy	0.02	0.01	1.02(1.01,1.04)	**0.001** ^**#**^	0.03	0.01	1.02(1.01,1.04)	**0.0008** ^**#**^
Self-HIV testing in lifetime								
Intercept	−1.82	0.43	–	–	−0.53	0.53	–	–
Social norm	0.11	0.03	1.12(1.07,1.18)	**< 0.001** ^**#**^	0.11	0.03	1.12(1.06,1.18)	**< 0.001** ^**#**^
Intercept	−0.39	0.39	–	–	0.95	0.49	–	–
Self-efficacy	0.02	0.02	1.03(0.99,1.07)	0.22	0.02	0.02	1.02(0.99,1.07)	0.24
Intercept	2.09	2.81	–	–	3.29	2.87	–	–
Social norm	−0.04	0.17	0.91(0.68,1.22)	0.54	−0.04	0.17	0.92(0.68,1.23)	0.56
Self-efficacy	−0.08	0.14	0.84(0.66,1.07)	0.16	−0.08	0.14	0.84(0.66,1.08)	0.17
Social norm*Self-efficacy	0.04	0.01	1.01(0.99,1.02)	0.16	0.04	0.01	1.01(0.99,1.02)	0.18

*Model adjusted for age (Continuous), marital status (Not married, engaged or Married, separated or widowed), education level (High school or below, college or bachelors, or masters or PhD) and monthly income (<1500 RMB, 1500–3000 RMB, 3001–5000 RMB, 5001–8000 RMB or >8000 RMB). # and bold indicates *P* < 0.05

Furthermore, univariate analysis indicated that MSM with higher sensitivity to the social norm was more likely to have had HIV testing at least once in their lifetime, though the difference was not significant (cOR = 1.02, [0.98–1.06]). Univariate analysis also revealed that MSM with higher self-efficacy score was more likely to have had HIV testing at least once in their lifetime (cOR = 1.09 [0.96–1.06]). We also detected an association of the interaction between sensitivity to HIV testing social norms and self-efficacy with lifetime HIV testing (cOR = 1.02 [1.01, 1.04], see Table 3).

Univariate analysis demonstrated that MSM with higher sensitivity to social norms had higher lifetime HIV self-testing in a lifetime (cOR =1.12 [1.07, 1.18]). MSM with higher self-efficacy score was more likely to have lifetime HIV self-testing in a lifetime, though the effect was not significant (cOR = 1.03, [0.99–1.07], see Table 3). Similar results were observed after adjusting for potential confounders (age, marital status, education level, and monthly income).

In our multivariate analysis, MSM with higher self-efficacy was more likely to have HIV testing in the past 3 months (adjusted odds ratio, aOR = 1.09 [1.05, 1.14]). For the increasing of every additional self-efficacy score, the HIV testing proportion in the past 3 months increased by 1.09 folds. Further, the interaction between social norm and self-efficacy was also associated with HIV testing in the past 3 months (aOR = 1.02 [1.01, 1.03]). MSM with higher self-efficacy and higher social norm were more likely to have HIV testing in the past 3 months.

In our multivariate analysis, MSM with higher self-efficacy and higher sensitivity to social norms had higher lifetime HIV testing proportion (aOR = 1.15 [1.11, 1.19] and 1.12 [1.06, 1.78], respectively). For every additional increase of self-efficacy score, the HIV testing proportion in the lifetime increased by 1.15 folds. For every additional increase of social norms score, the HIV testing proportion in the lifetime increased by 1.12 folds. Further, the interaction effect between social norm and self-efficacy was also associated with lifetime HIV testing (aOR = 1.02 [1.01, 1.04], see Table 3). MSM with higher self-efficacy and higher social norm were more likely to have HIV testing in the life time.

## Discussion

Previous studies have shown that HIV testing self-efficacy and social norms had an impact on the uptake of HIV testing. This study extends the existing literature by assessing the relationship between HIV testing and social norms, self-efficacy, and the potential interaction between social norms and self-efficacy.

Our study showed that sensitivity to HIV testing social norms was positively associated with having received an HIV testing within the past 3 months as well as lifetime HIV testing. This is consistent with the findings of previous studies from northern Nigeria and rural Uganda [7]. This finding supports the hypothesis that improving HIV testing social norms can potentially improve the HIV testing uptake among Chinese MSM. Intervention packages aimed at improving HIV testing proportion should consider social norms and perceptions of social norms as an actionable part of the overall intervention strategies.

Our study also indicated that HIV testing self-efficacy was positively associated with lifetime HIV testing. This finding is consistent with previous studies, which have shown that improving HIV testing self-efficacy is useful for promoting HIV testing and medication adherence [18–20]. Our study suggests the importance of developing strategies (i.e. raising the severity perception [14]) to strengthen MSM self-efficacy, thereby improving safe sexual behavior and HIV testing proportion [21]. More importantly, integrating self-efficacy with other ongoing interventions (i.e., HIV self-testing), and further improving the effectiveness of these implementation strategies will be more practical.

Further, our study demonstrated that sensitivity to social norms and self-efficacy interact with each other in promoting HIV testing, and MSM with both higher sensitivity to social norms and self-efficacy were more likely to have HIV testing. This finding is consistent with previous literature that positive social norms and self-efficacy would work together in improving condom use among MSM [22]. There are several potential reasons for this phenomenon. First, MSM with higher sensitivity to social norms may be more likely to discuss HIV testing with friends [23]. This kind of interaction may further increase their self-efficacy and lead to HIV testing. Second, reasoned action theory has shown that when people are aware of their community’s support for an act, they will be more likely to carry out such behavior [24]. Third, according to social cognitive theory, people with higher self-efficacy and sensitivity to social norms may make a greater effort to accomplish their goals in the near future [15]. Further implementation studies that can combine the social norms and self-efficacy strategies, and working together to improve HIV testing together would be more effective.

In our study, the prevalence of HIV testing among MSM remained low with 32.6% of participants having been tested for HIV in the last 3 months and 62.5% of participants having been tested for HIV in their lifetime. These testing proportions are higher than those from Thailand in 2014 [25] and Zhejiang, China in 2014 [1], but lower than those from Cambodia in 2015 [26]. The HIV testing proportion is still far behind the UNAIDS target for 90% testing among infected individuals in 2014 [27]. This may be attributable to many factors. First, there is low awareness among MSM of the infection risk associated with sexual behavior [28]. Secondly, even MSM who are willing to be tested often need to travel to other cities for testing in order to avoid social stigma [29]. Thirdly, younger MSM are less likely to have been tested for HIV, potentially due to their fear of HIV testing in healthcare settings [30], in our study about 54.4% of the participants were 25 years old or younger. Lastly, the geographical distribution of HIV testing sites is unbalanced in China [31].

This study has several limitations. Firstly, we recruited participants through a mobile dating application, so participants tended to be younger, more highly educated, with a higher burden of syphilis and HIV [32]. MSM in remote rural areas in China were not included in the study. Secondly, HIV testing in the past 3 months was self-reported, which might lead to social desirability bias. Thirdly, the Cronbach’s α of HIV testing social norm and self-efficacy were a bit low. Lastly, like many other online cross-sectional studies, there might be a selection bias in the study, as the online participants are tended to be young and well educated [33].

## Conclusions

In summary, our results showed that the prevalence of HIV testing among MSM in China is suboptimal and may result in continuing transmission of HIV. However, most of the MSM who participated in this study were fairly confident in their likelihood of having HIV testing. Our study noted the association of the interaction between sensitivity to HIV testing-related social norms and self-efficacy with HIV testing and self-HIV testing among Chinese MSM. MSM with higher sensitivity to social norms and self-efficacy were more likely to have HIV testing. Future studies should investigate how social norms and self-efficacy are working together in promoting HIV testing. Policies and intervention packages (such as culturally competent sexual health education interventions [34]) should focus on increasing positive social norms and self-efficacy among MSM as an essential component of the overall intervention strategy.

## Additional file

Additional file 1: Semiparametric regression map for social norm and self-efficacy. (TIFF 351 kb)

## Abbreviations

aOR: Adjusted odds ratio
cOR: Crude odds ratio
DAG: Directed acyclic graph
HIV: Human Immunodeficiency Virus
IQR: Interquartile range
MSM: Men who have sex with men
PITC: Provider-initiated testing and counseling
PLWH: People living with HIV
VCT: Voluntary counseling and testing
