# Supplementary figures and images for: The interaction between HIV testing social norms and self-efficacy on HIV testing among Chinese men who have sex with men: results from an online cross-sectional study
Source: BMC Infect Dis. 2018 Oct 30;18:541. doi: 10.1186/s12879-018-3454-5 (PMC6208016; doi:10.1186/s12879-018-3454-5)

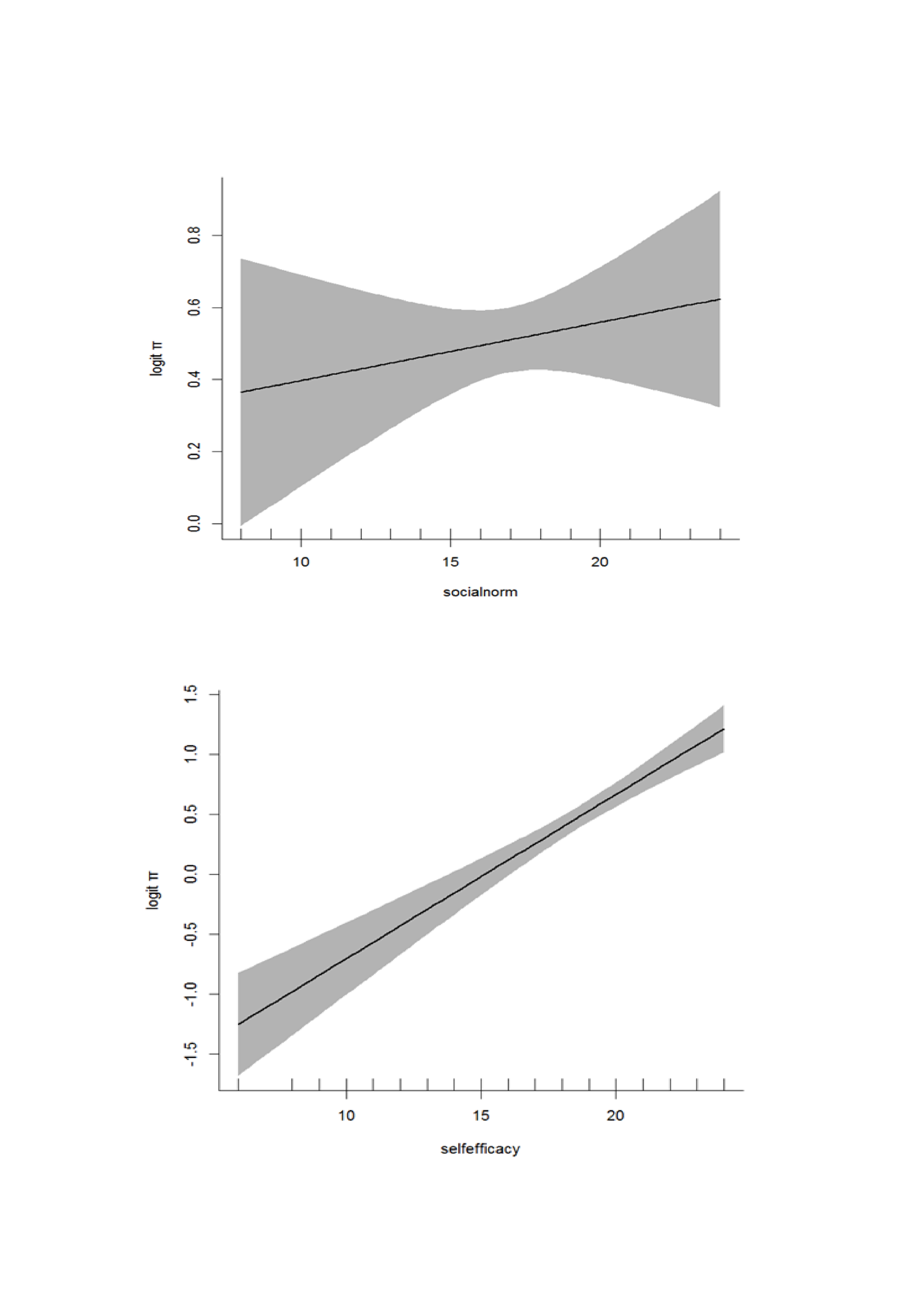

Supplement: Supplementary file 1 — Semiparametric regression map for social norm and self-efficacy. (TIFF 351 kb) [file 12879_2018_3454_MOESM1_ESM.tiff]
